# Preparation and Efficacy of Newcastle Disease Virus DNA Vaccine Encapsulated in PLGA Nanoparticles

**DOI:** 10.1371/journal.pone.0082648

**Published:** 2013-12-26

**Authors:** Kai Zhao, Wei Li, Tingting Huang, Xiaomei Luo, Gang Chen, Yang Zhang, Chen Guo, Chunxiao Dai, Zheng Jin, Yan Zhao, Hongyu Cui, Yunfeng Wang

**Affiliations:** 1 Laboratory of Microbiology, College of Life Science, Heilongjiang University, Harbin, China; 2 Division of Avian Infectious Diseases, State Key Laboratory of Veterinary Biotechnology, Harbin Veterinary Research Institute, CAAS, Harbin, China; 3 Key Laboratory of Chemical Engineering Process and Technology for High-efficiency Conversion, Heilongjiang University, Harbin, China; Federal University of São Paulo, Brazil

## Abstract

**Background:**

Although the Newcastle disease virus (NDV) inactivated vaccines and attenuated live vaccines have been used to prevent and control Newcastle disease (ND) for years, there are some disadvantages. Recently, newly developed DNA vaccines have the potential to overcome these disadvantages. The low delivery efficiency, however, hindered the application of DNA vaccines for ND in practice.

**Methodology/Principal Findings:**

The eukaryotic expression plasmid pVAX1-F (o) DNA that expressed the F gene of NDV encapsulated in PLGA nanoparticles (pFNDV-PLGA-NPs) were prepared by a double emulsion-solvent evaporation method and optimal preparation conditions of the pFNDV-PLGA-NPs were determined. Under the optimal conditions, the pFNDV-PLGA-NPs were produced in good morphology and had high stability with a mean diameter of 433.5±7.5 nm, with encapsulation efficiency of 91.8±0.3% and a Zeta potential of +2.7 mV. Release assay *in vitro* showed that the fusion gene plasmid DNA could be sustainably released from the pFNDV-PLGA-NPs up to 93.14% of the total amount. Cell transfection test indicated that the vaccine expressed and maintained its bioactivity. Immunization results showed that better immune responses of SPF chickens immunized with the pFNDV-PLGA-NPs were induced compared to the chickens immunized with the DNA vaccine alone. In addition, the safety of mucosal immunity delivery system of the pFNDV-PLGA-NPs was also tested in an *in vitro* cytotoxicity assay.

**Conclusions/Significance:**

The pFNDV-PLGA-NPs could induce stronger cellular, humoral, and mucosal immune responses and reached the sustained release effect. These results laid a foundation for further development of vaccines and drugs in PLGA nanoparticles.

## Introduction

Newcastle disease (ND) is a highly contagious viral disease of poultry and is characterized by nervous, respiratory, enteric and reproductive infection [1]. The causative agent of ND is virulent Newcastle disease virus (NDV) [2], [3]. NDV is a single stranded, non-segmented, enveloped RNA virus with negative polarity and consists of six genes that code for RNA polymerase (L gene), haemagglutinin-neuraminidase (HN gene), fusion (F gene), matrix (M gene), phosphoprotein (P gene) and nucleocapsid (NP gene) proteins [4]. Among these genes, F gene encodes an indispensable glycoprotein that allows the binding and fusion of NDV to the host cells to initiate ND and induces vaccine immunity, and has a better immunogenicity and more advantages compared with HN protein [5].

Although the inactivated and attenuated live NDV vaccines have been playing an important role in prevention and control of ND in practice, these conventional vaccines have some disadvantages including partial virus toxicity reservation, induction of respiratory pathological changes, and difficulty of differentiation between vaccine immunization and natural infection. DNA-based vaccines have been proven to induce long-lived cellular and humoral immune responses in both humans and animals [6]–[8]. To date, DNA vaccines are administered as either aqueous solutions or freeze-dried powders. However, DNA vaccines have high hydrophilicity and a low efficiency of distribution between oil and water phase [9], [10]. After intramuscular injection, it is difficult for the vaccines to move through cell membranes, so only a small amount of vaccines reaches antigen-presenting cells (APCs) to induce immune responses [11]. Low levels of DNA vaccine expression and weak immune responses [12]–[14], especially in large animal models [15], have limited the clinical applications of these novel DNA vaccines. As indicated in the previous studies [16], [17], the need for much lower doses of plasmid DNA and more effective delivery systems that would improve the transfection efficiency *in vivo* is pressing. Therefore, it is necessary to develop a suitable DNA vaccine delivery system for mass vaccination in farms, and a number of new techniques have recently been developed to introduce foreign DNA into cells.

Among all the available antigen delivery systems [18]–[20], the biodegradable materials used in mucosal immune delivery system have the characteristics of ease to be digested and absorbed by the organism. They not only have biodegradability, biocompatibility, low toxicity, good biological characteristics, and ease to be modified into new drug carrier that can be administered by different routes, but also protect antigen and DNA from damage. Based on these characteristics, the biodegradable materials have attracted much attention and have many applications in encapsulating a wide range of bioactive agents including proteins and peptides. Current researches on cationic Poly (D, L)-lactic-co-glycolic acid (PLGA) and chitosan focus on their use as a novel delivery carrier, target delivery and tissue engineering applications [21], [22].

PLGA microparticles or nanoparticles have the potential to act as mediators of DNA transfection targeting phagocytic cells such as macrophages, protect against biological degradation by nucleases [22], [23] and enhance drug or plasmid DNA long-acting release and expression [24], [25]. As an efficient protein/peptide/gene drug delivery carrier, PLGA nanoparticles can help drugs better permeate through the different biological barriers such as the blood brain barrier [26], [27], gastrointestinal mucosa [28]–[31], nasal mucosa [32], [33] and retina [34]. The PLGA nanoparticles could avoid lysosome, enter the cytoplasm, and release the plasmid DNA [35], [36]. In addition, the drug-loaded PLGA nanoparticles can be presented by antigen-presenting cells such as macrophages and dendritic cells to specific T cells to induce cell-mediated immunity [37]. Rajapaksa et al. (2010) reported that the PLGA nanoparticles could improve the ability of microfold cell (M cell) endocytosis, which laid the foundation for nanoparticles to be used as a mucosal immune delivery system [38].

In this study, the eukaryotic expression plasmid pVAX1-F (o) DNA encapsulated in PLGA nanoparticles were prepared and the immune responses elicited in SPF chickens immunized with the PLGA-plasmid DNA nanoparticles were evaluated. In addition, bioactivity and safety of the PLGA nanoparticles were studied by *in vitro* transfection and cytotoxicity analyses. This work provided a new insight into the study of gene vaccines and gene therapies, and the plasmid DNA loaded PLGA-NPs demonstrated to be a newly developed drug release carrier system with immense potential for medical applications.

## Materials and Methods

### Ethics Statement

Care of laboratory animals and animal experimentation were performed in accordance with animal ethics guidelines and approved protocols. All animal studies were approved by the Animal Ethics Committee of Harbin Veterinary Research Institute of the Chinese Academy of Agricultural Sciences (CAAS) and the Animal Ethics Committee of Heilongjiang Province (SYXK (H) 2006-032).

### Optimization of the pFNDV-PLGA-NPs preparation conditions

The eukaryotic expression plasmid pVAX1-F (o) DNA that expressed the F gene of NDV was provided by State Key Laboratory of Veterinary Biotechnology, Harbin Veterinary Research Institute, CAAS. The PLGA- plasmid DNA nanoparticles (pFNDV-PLGA-NPs) were prepared by a water/oil/water (w/o/w) double emulsion-solvent evaporation method [39]. The orthogonal experimental design with three factors and four levels is shown in Table 1, and the encapsulation efficiency was used as an indicator.

**Table 1:** Optimization of the pFDNA-PLGA-NPs preparation conditions.

Experiment No	A PLGA concentration (mg/ml)	B Plasmid DNA/PLGA (%, w/w)	C PVA concentration (%, w/v)	Encapsulation efficiency (%)
1	20	0.5	0.5	84.2
2	20	1.0	1.0	80.6
3	20	1.5	2.0	82.4
4	20	2.0	3.0	77.8
5	30	0.5	1.0	86.1
6	30	1.0	0.5	79.5
7	30	1.5	3.0	86.3
8	30	2.0	2.0	81.7
9	40	0.5	2.0	92.1
10	40	1.0	3.0	75.4
11	40	1.5	0.5	87.8
12	40	2.0	1.0	82.4
13	50	0.5	3.0	84.0
14	50	1.0%	2.0	82.9
15	50	1.5	1.0	81.8
16	50	2.0%	0.5	83.2
T1	81.25%	88.35%	84.18%	
T2	83.40%	79.60%	82.73%	
T3	84.93%	85.08%	84.78%	
T4	84.73%	81.28%	82.63%	
R	3.68%	8.75%	2.15%	

Note: K_1_, K_2_ and K_3_ represent the mean value of factors at 1, 2 and 3 levels, respectively. R represents the variation range among T1 to T4.

### Stability of the plasmid DNA in the pFNDV-PLGA-NPs

To test stability of the pFNDV-PLGA-NPs, naked plasmid DNA (3.0 µg/µl of 20% of Na_2_SO_4_) and the pFNDV-PLGA-NPs suspension (10 µl, equivalent to 3.0 µg plasmid DNA) were incubated with 50 units of DNase I at 37°C in 50 µl of reaction buffer (50 mmol/l KCl, 10 mmol/l Tris-HCl, pH 9.0, 10 mmol/l MgCl_2_, and 0.1% Triton X-100) for 30, 60, 120 and 180 min, respectively. The reaction was stopped by adding 100 µl termination solutions (400 mmol/l NaCl, 100 mmol/l EDTA, pH 8.0) at 65°C for 10 min. Then 4.0 µl lysozyme (0.2 U/ml) was added at 37°C water bath for 4 h. The integrity of the plasmid DNA which been taken in the different times was analyzed using 1.0% agarose gel electrophoresis.

### *In vitro* expression of the pFNDV-PLGA-NPs

The transfection experiment and Western blotting analyses for *in vitro* expression of the plasmid pVAX1-F (o) from the pFNDV-PLGA-NPs in BHK cells were performed as described previously [22].

### *In vitro* transfection of pFNDV-PLGA-NPs

BHK cells were grown in 6-well plates with proper density and cultured at 37°C in a 5% CO_2_ incubator. Cell populations with 70–80% viability were used for in vitro transfection assay. The medium was discarded and the cells washed twice with Dulbecco's minimum essential medium (DMEM, Gibico, USA) without antibiotics and serum. The plasmid DNA was extracted from the pFNDV-PLGA-NPs and naked plasmid DNA used as a positive control. The transfection experiment was carried out according to the instructions from the LipofectamineTM 2000 reagent kit (Invitrogen, USA). Meanwhile, blank PLGA-NPs and BHK cells were used as the negative controls. All transfection experiments were performed in triplicate. An indirect immunofluorescent test was used to monitor the expression of plasmid DNA in transfected cells. The NDV positive serum (HVRI) and fluorescein isothiocyanate-labeled goat-anti-chicken IgG (Sigma, St. Louis, MO, USA) were diluted at 1∶100 and 1∶5000, respectively.

### Western blot analysis for expression of pFNDV-PLGA-NPs

After 72 h of transfection, the BHK cells were collected and disrupted using RIPA solution (50 mmol/l Tris-HCl (pH 8.5), 5 mmol/l 2-Hydroxy-1-ethanethiol, 100 mmo1/l KCl, l mmol/l PMSF, 1% Nonidet®P-40). The lysate was centrifuged at 14000 r/min at 4°C, and the supernatant were collected and stored at −80°C The supernatant was mixed with 2×SDS-PAGE loading buffer (100 mmol/l Tris-HCl (pH 6.8), 0.2% bromophenol blue, 20% glycerol, 200 mmol/l DTT) and boiled for 5 min. After cooling to room temperature, 20 µl of samples were loaded onto a 10% SDS-PAGE gel. After electrophoresis, proteins were transferred to a nitrocellulose membrane (Amersham, Sweden) using a BioRad semi-dry unit. The membrane was washed with PBS and blocked with 5% fat free milk overnight, followed by incubation with a NDV positive serum at a 1∶500 dilution for 1 h. After washing with PBST for three times, FITC labeled goat-anti-chicken secondary antibody was added at a dilution of 1∶5000 for 1 h; and the image was acquired using an Odyssey infrared imaging system (LI-COR Odyssey, USA).

### Evaluation of the safety of pFNDV-PLGA-NPs

#### *In vitro* cytotoxicity of the pFNDV-PLGA-NPs

*In vitro* cytotoxicity of the pFNDV-PLGA-NPs was evaluated as described previously [22]. BHK cells were cultured in DMEM (containing 10% fetal bovine serum) and then diluted to 2×10^6^/ml. Cells were transferred to 96-well plates at 200 µl per well and cultured at 37°C for 5 h. Fifty microliters of pFNDV-PLGA-NPs (diluted in DMEM culture at 1 mg/ml) were added into the wells, followed by incubation at 37°C for 2 h. Cell culture medium was used as a positive control for cell viability. Ten microliters of WST-8 reagent (Dojindo, Japan) was added and incubated for 5 h. OD450 was measured to determine survival rate of the cells, which was calculated using the following formula: Survival rate (%) = [(As-Ab)/(Ac-Ab)]×100%. The As represents the test wells (containing cell medium, WST-8 and pFNDV-PLGA-NPs); Ac represents the control wells (containing cell medium and WST-8); and Ab represents the blank wells (cell medium no containing pFNDV-PLGA-NPs and BHK cells, containing WST-8). WST-8 is reduced by dehydrogenases in cells to obtain a yellow colored product (formazan), which can be directly used for cytotoxicity assay.

#### Safety of the pFNDV-PLGA-NPs

Thirty 4-week-old SPF chickens obtained from Harbin Veterinary Research Institute Laboratory Animal Center were grouped randomly into two groups: chickens in Group 1 were immunized intranasally (i.n.) with 0.2 ml of the pFNDV-PLGA-NPs; chickens in Group 2 were immunized intramuscularly (i.m.) with 0.2 ml of the naked plasmid DNA. Any abnormal changes in the immunized chickens were continuously observed and recorded for three weeks.

### Immunization of SPF chickens

One hundred and seventy five 14-day-old SPF chickens were grouped randomly into seven groups (25 chickens per group). Chickens in Group 1 were immunized i.m. with PBS; Chickens in Group 2 were immunized i.m. with the blank PLGA-NPs; Chickens in Group 3 were immunized i.n. with the blank PLGA-NPs; Chickens in Group 4 were immunized i.m. with the naked plasmid DNA (200 µg); Chickens in Group 5 were immunized i.m. with the pFNDV-PLGA-NPs (containing 200 µg plasmid DNA); Chickens in Group 6 were immunized i.n. with the pFNDV-PLGA-NPs (containing 200 µg plasmid DNA); Chickens in Group 7 were immunized with the pFNDV-PLGA-NPs (containing 200 µg plasmid DNA) i.n. and i.m.. The primary immunization and the booster immunization were administered i.n. and i.m. with same dose, respectively.

### IgG antibody detection in serum by enzyme linked immunosorbent assay (ELISA)

Blood was collected from the wing vein at 7, 14, 21, 28, 35, 42 and 49 d post the first immunization, and the serum separated by centrifugation at 2200 r/min for 10 min at 4°C. ELISA was performed to assess the titers of the specific IgG in immune sera using the NDV IgG ELISA Kit (Rapidbio Co. Ltd, West Hills, CA, USA) according to the manufacturer's instructions.

### IgA antibody assay

Tears, tracheal fluid, bile, serum were collected from two chickens euthanized once a week after the first immunization to evaluate the mucosal immune response. IgA antibody was detected by using NDV IgA ELISA Kit (Rapidbio Co. Ltd, USA) according to the manufacturer's instructions.

### Proliferation of lymphocytes from immunized chickens

Lymphocyte proliferation of the immunized chickens was carried out using MTT colorimetric assay as previously described [22]. The spleen of 3 immunized chickens was removed aseptically at the 13 d and 27 d after the first immunization. The spleen was filtered through 200 micron copper mesh and single cell suspensions were prepared from the filtrate. Erythrocytes were lysed using 0.75% Tris-NH_4_Cl (pH 7.4). Spleen cells were suspended in RPMI 1640 medium containing 10% fetal bovine serum, and diluted to 2×10^7^ cells/ml. Cell suspensions were transferred to 96-well plates at 200 µl per well. Twenty microliters of purified and inactivated NDV was used as specific stimulating antigen. Wells containing 75 µg/ml Con A were used as positive controls, and those without stimulating antigen were used as negative controls. All the cells were cultured at 5% CO_2_ and 37°C for 60 h, then 10 µl WST-8 (2-(2-methoxy-4-nitrophenyl)-3-(4-nitrophenyl)-5-(2,4-disulfophenyl)-2H-tetrazolium, monosodium salt) was added into each well followed by culturing for another 5 h. The OD450 was measured to determine the stimulation index (SI). All experiments were repeated three times and each was measured in triplicate. The OD450 was measured to determine the stimulation index using the following formula:

The stimulation index (SI) is expressed with average OD value in the test group divided by average OD value in negative controls.

### Statistical analysis

All experiments were repeated three times and each measured in triplicate. Data were presented as mean values ± standard deviation. Mean values were analyzed using the one-sided Student's t-test. Differences were considered to be statistically significant at *p*<0.05.

## Results

### Preparation of the pFNDV-PLGA-NPs

As shown in Table 1, the factors that affected the pFNDV-PLGA-NPs preparation were ranked from high to low impact: the plasmid DNA/PLGA ratio > PLGA concentration > PVA concentration. The optimal combination for the pFNDV-PLGA-NPs preparation was 0.5% ratio of the DNA to PLGA, 40 mg/ml PLGA and 2.0% PVA. A validation test was conducted according to the optimal combination described above; EE was 91.8±0.3%, higher than any EE combination in the orthogonal experiment (Table 1). The final optimal condition for the pFNDV-PLGA-NPs was the primary emulsion (50 w, 30 s), the secondary emulsion (50 w, 60 s), and 0.5% ratio of the DNA to PLGA and 40 mg/ml PLGA with 2.0% PVA.

### Characterization of the pFNDV-PLGA-NPs

The pFNDV-PLGA-NPs had regular round shape and good dispersion, but did not have aggregation or subsidence damage (Fig. S1). These particles were measured by a Zeta Sizer 2000 from Malvern Instruments (Southborough, MA, USA) and the average particle size was 433.5±7.5 nm. The particle polydispersity index was 0.41 with a zeta potential of +2.7 mV (Fig. S2 A and B)

### Stability of plasmid DNA in the pFNDV-PLGA-NPs

**Figure 1:**
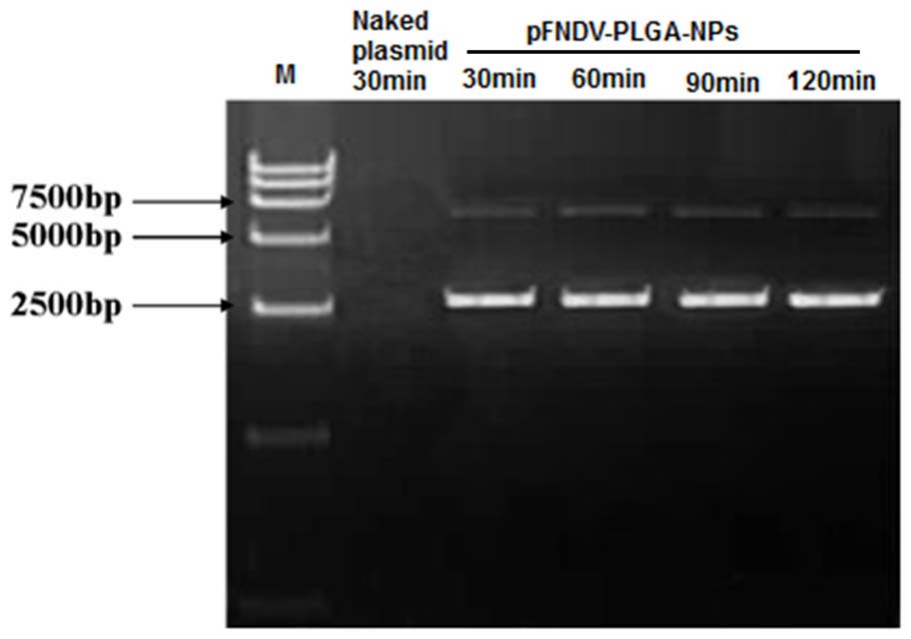
Stability analysis of the plasmid DNA after encapsulation in the PLGA nanoparticles. Lane 1: DL 15000 marker; Lane 2: the naked plasmid DNA treated by DNase I for 30 min; Lane 3: the pFNDV-PLGA-NPs treated by DNase I for 30 min; Lane 4: the pFNDV-PLGA-NPs treated by DNase I for 60 min; Lane 5: pFNDV-PLGA-NPs treated by DNase I for 120 min; Lane 6: the pFNDV-PLGA-NPs treated by DNase I for 180 min.

The naked plasmid DNA was degraded by incubation with DNase I for 30 min (Lane 2 of Fig. 1). However, the plasmid DNA encapsulated in the pFNDV-PLGA-NPs was protected from degradation by DNase I (Fig. 1). These results demonstrated that PLGA encapsulation could protect the plasmid DNA from DNase I digestion.

### *In vitro* expression of the pFNDV-PLGA-NPs

**Figure 2:**
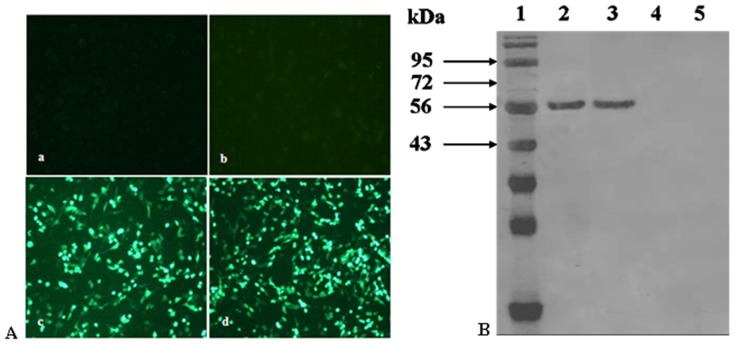
*In vitro* expression of the pFNDV-PLGA-NPs in BHK cells. (A) by the indirect immunofluorescence analysis (×100). (a) BHK cell group as the negative control; (b) blank PLGA-NPs group; (c) the naked plasmid DNA pVAX1-F (o) group; (d) the plasmid DNA from pFNDV-PLGA-NPs transfected group. (B) by Western blotting. Lane 1: protein marker; Lane 2: the naked plasmid DNA groups; Lane 3: the plasmid DNA from pFNDV-PLGA-NPs transfected group; Lane 4: blank PLGA-NPs group; Lane 5: BHK cells group as the negative control.

As shown in Fig. 2A, specific fluorescence was observed in the BHK cells transfected with plasmid DNA from the pFNDV-PLGA-NPs and the naked plasmid DNA, but the blank PLGA-NPs group and the cell control group had no observed fluorescence. The antigen expression was further confirmed by Western blotting (Fig. 2B), indicating that the plasmid DNA encapsulated in the pFNDV-PLGA-NPs and the naked plasmid DNA could express the expected 58 kDa antigen in BHK cells, but blank PLGA-NPs and cell control group had no expressed antigen.

### Evaluation of the safety of pFNDV-PLGA-NPs

#### In vitro cytotoxicity analysis of the pFNDV-PLGA-NPs

*In vitro* cytotoxicity of the pFNDV-PLGA-NPs was evaluated and the survival rate was 80.14±8.27%. No significant changes in cell morphology were observed in comparison to control cells. These results showed that the pFNDV-PLGA-NPs had low cytotoxicity.

#### In vivo cytotoxicity analysis of the safety of pFNDV-PLGA-NPs

Chickens in two groups immunized with either the pFNDV-PLGA-NPs or the naked plasmid DNA i.n. had no nervous signs, no clinical symptoms and no necropsy lesions within 3 weeks post the inoculation. No obvious abnormal changes were observed in these immunized chickens. These results revealed that the pFNDV-PLGA-NPs were safe by the tested administration routes.

### Immune efficacy of the pFNDV-PLGA-NPs

#### IgG antibody in serum

**Figure 3:**
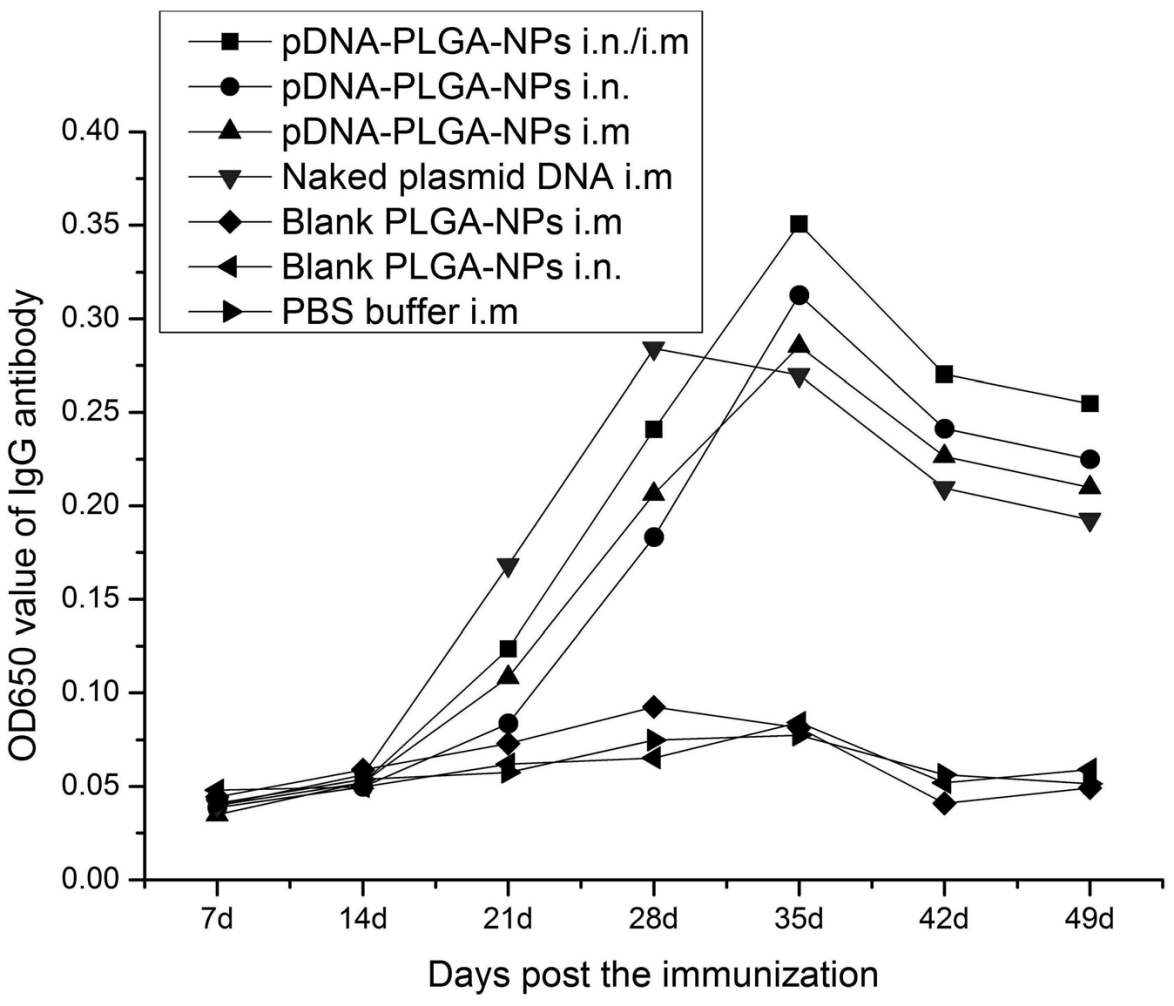
IgG antibody titers in serum of SPF chickens immunized with PBS (i.m.), blank PLGA-NPs (i.m.), blank PLGA-NPs (i.n.), and the naked plasmid DNA (i.m.), pFNDV-PLGA-NPs (i.m.), pFNDV-PLGA-NPs (i.n.) or pFNDV-PLGA-NPs (i.m./i.n.). IgG antibody titers were presented as mean ± SD of five experiments. Data with different small letters show significant difference (*p*<0.05).

As shown in Fig. 3, the antibody titers were detected at a lower level at first 2 weeks after the immunization in each of the immunization groups. The antibody titers quickly increased 3 weeks post immunization in chickens immunized with the pFNDV-PLGA-NPs i.n. and i.n./i.m., peaked at 5 weeks post immunization. The antibody titers of chickens immunized with the naked plasmid DNA i.m. increased from the third week and peaked at the fourth week post immunization, and had extremely significant differences (*p*<0.01) compared with chickens in the other immunization groups. The chickens immunized with the pFNDV-PLGA-NPs i.n. and i.n./i.m. maintained a higher IgG level up to the seventh week post immunization, and the antibody titers were significantly different from those of chickens immunized with the naked plasmid DNA i.m. (*p*<0.01), suggesting a sustained release of the DNA vaccine. These results indicated that the pFNDV-PLGA-NPs could release DNA vaccine slowly and continuously and stimulate specific B lymphocytes, thereby increasing serum antibody titers.

#### IgA antibody assay

**Figure 4:**
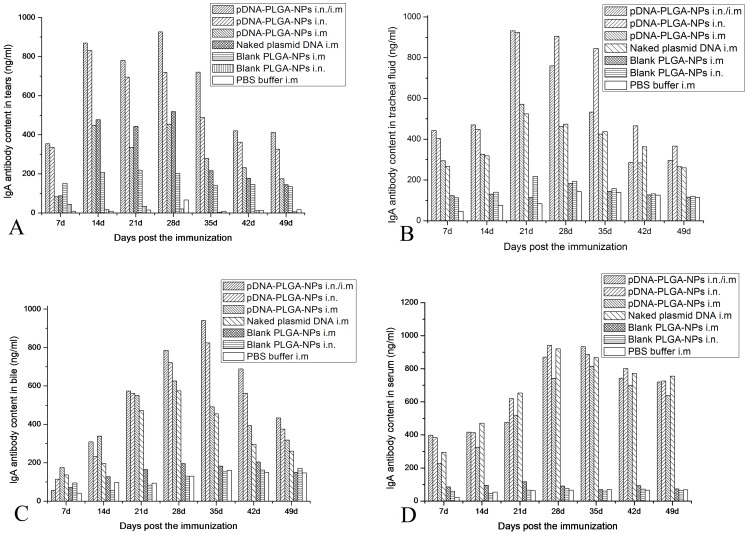
IgA antibody content in tears of SPF chickens immunized with PBS (i.m.), blank PLGA-NPs (i.m.), blank PLGA-NPs (i.n.), and the naked plasmid DNA (i.m.), pFNDV-PLGA-NPs (i.m.), pFNDV-PLGA-NPs (i.n.) or pFNDV-PLGA-NPs (i.m./i.n.). (A) IgA antibody content in tears; (B) IgA antibody content in tracheal fluid; (C) IgA antibody content in bile; (D) IgA antibody content in serum. IgA antibody content was presented as mean ± SD of five experiments. Data with different small letters show significant difference (*p*<0.05).

The changes of IgA content in tears, tracheal fluid, bile and serum are shown in Fig. 4. Chickens immunized with the pFNDV-PLGA-NPs i.n. and the combined immunization routes had significantly higher IgA antibody titers (*p*<0.01) and longer IgA antibody secretion period in tears (Fig. 4A), tracheal fluid (Fig. 4B) and bile (Fig. 4C) compared with the blank PLGA-NPs and PBS buffer control groups. Although there was no difference of the IgA antibody titers in serum among the immunization groups, the IgA antibody titers in these groups were significantly higher than those of the blank PLGA-NPs and PBS buffer control groups (Fig. 4D). These findings indicated that the pFNDV-PLGA-NPs induced quicker and better mucosal immune responses than the naked plasmid DNA vaccine.

#### Lymphocyte proliferation assay

As shown in Table 2, no significant differences were found between chickens immunized with the pFNDV-PLGA-NPs i.m., i.n. or i.n./i.m. and chickens immunized with the plasmid DNA i.m. (*p*>0.05) at the second week post immunization. However, by the fourth week post immunization, the stimulation indices of chickens immunized with the pFNDV-PLGA-NPs either i.m., i.n. or i.n./i.m. were extremely significantly higher than those of chickens immunized with the plasmid DNA, or with blank PLGA-NPs (*p*<0.01). The chickens immunized with the pFNDV-PLGA-NPs either i.n. or i.n./i.m. had significantly higher stimulation indices than chickens immunized with the pFNDV-PLGA-NPs i.m. (*p*<0.05), but no significant differences were found between chickens immunized with the pFNDV-PLGA-NPs i.n and i.n./i.m. (*p*>0.05). These findings indicated that the pFNDV-PLGA-NPs significantly enhanced immune function of T lymphocytes in the immunized chickens.

**Table 2:** The stimulating index of T lymphocyte proliferation in SPF chickens immunized with either pFDNA-PLGA-NPs, pVAX1-F (o), or Blank NP.

Groups	Days post the immunization	
	14	28
pFDNA-PLGA-NPs i.n./i.m	1.996±0.262^a^	3.183±0.137^Aa^
pFDNA-PLGA-NPs i.n.	1.878±0.237^a^	3.077±0.177^Aa^
pFDNA-PLGA-NPs i.m	1.730±0.230^a^	2.856±0.145^Bb^
pVAX1-F (o) i.m	1.844±0.234^a^	2.335±0.199^Cc^
Blank PLGA-NPs i.n.	1.049±0.307^b^	1.032±0.137^Dd^
Blank PLGA-NPs i.m	0.985±0.244^b^	1.085±0.127^Dd^

Values are presented as mean ± S.D. of five experiments in each group. Values within the same column with the different lower case letter in the superscript are significantly different (*p*<0.05; Student's t-test), and with the different upper case letter in the superscript are extremely significant different (*p*<0.01; Student's t-test). The stimulation indices of chickens immunized with the pFDNA-PLGA-NPs by either i.m, i.n. or i.n./i.m. were extremely significantly higher than those of chickens immunized with the plasmid DNA pVAX1-F (o), or with blank PLGA-NPs (*p*<0.01), and the chickens immunized with the pFDNA-PLGA-NPs either i.n. or i.n./i.m. had significantly higher stimulation indices than the chickens immunized with the pFDNA-PLGA-NPs i.m at the fourth week post immunization (*p*<0.05).

## Discussion

The nasal mucosa is an important arm of the mucosal immune system and plays an important role in preventing infectious diseases since it is the first portal of entry for inhaled antigens and pathogens. The mucous membrane of the digestive tract and respiratory tract are vulnerable to direct stimulation by antigen, so as to achieve a high level of antibodies against NDV. In addition, because the transmission route of NDV is mainly the respiratory tract and digestive tract, mucosal immune response plays an important role in ND prevention and control, especially the nasal mucosa that can lead to both efficient mucosal and systemic immune responses [40].

Many factors in the nanoparticle preparation process, such as ultrasonication and high agitation speed, can lead to DNA degradation and even loss of bioactivity. Therefore, keeping DNA stability is particularly important in preparation of DNA vaccines. In this study, measures such as reducing the ultrasonic power, shortening the ultrasonic time and ultrasound in an ice bath were used to reduce the DNA degradation in the preparation process. Finally, we standardized the optimal preparation conditions of the nanoparticles with low ultrasonic power at 50 w and ultrasonic times at 30 s for the primary emulsion, and ultrasonic power at 50 w and ultrasonic times at 60 s for the secondary emulsion. In addition, based on our previous study [39], the orthogonal experiments with three factors and four levels were designed, and the optimized conditions were 40 mg/ml PLGA, 200 µg plasmid, and 2% PVA. A previous study showed that nanoparticles with high surface Zeta potential could combine plasmid DNA better and slow down the burst release process [41]. The pFNDV-PLGA-NPs in this study also protected the plasmid DNA encapsulated in the PLGA nanoparticles from DNase I digestion. These results in this study showed that the prepared pFNDV-PLGA-NPs met desired objectives/requirements and provided a theoretical basis for DNA vaccine nanoparticle preparation.

Another critical feature of DNA loaded nanoparticle vaccines is the slow release of antigen DNA that provides long term antigen stimulation [42]. In this study, the pFNDV-PLGA-NPs slowly released the loaded DNA continuously for up to 16 days from the *in vitro* release analysis that was conducted under the physiological pH conditions. There was a burst release of the plasmid DNA between 0 h and 48 h due to detachment of the plasmid DNA that was adhered to the nanoparticles surface. From days 2 to 10, the plasmid DNA was continuously released at a high level, which was the main and stable stage of the nanoparticle release (More details were described in Materials and Methods S1). A previous study has shown that the high concentration of the plasmid DNA trapped inside the nanoparticles could accelerate the plasmid DNA release by forming holes on the surface of the nanoparticles [43].

The other important criteria are the bioactivity and safety of the new developed pFNDV-PLGA-NPs vaccine. They were tested both *in vitro* and *in vivo* analyses in our study, and the results showed that the nanoparticle production procedure was safe, and the bioactivity of the plasmid DNA was kept after the production of the nanoparticles.

The newly developed pFNDV-PLGA-NPs could induce strong cellular and humoral immune responses. The quick and strong release of secretory IgA, the main factor of mucosal immune response, is critical for NDV prevention because the transmission route of ND is mainly the respiratory and digestive tracts. The nasal mucosa is the first portal of entry for inhaled antigens and can lead to both efficient mucosal and systemic immune responses [40]. Although the IgA plays a key role in preventing the infection, IgA can only last for a short period of time as IgA has a short half-life and can decompose quickly. Therefore, the humoral immune response is very important, which lasts for a much longer time. IgG is a very important antibody in the humoral immune response of chickens. Our results showed that a high level of IgG antibodies was detected in the pFNDV-PLGA-NPs i.n. group and the i.n./i.m. combined group. Although the IgG level peaked 1 week late than the DNA plasmid i.m., the immunized chickens maintained the high level of IgG for a longer time than those by the i.m. DNA vaccine injection. Most importantly, chickens in the pFNDV-PLGA-NPs i.n. group and the i.n./i.m. combined group had strong mucosal immune response, which was not detected in the chickens immunized with DNA plasmid i.m.. In summary, SPF chickens immunized with the pFNDV-PLGA-NPs induced stronger humoral immunity and reached the sustained release effect. The serum specific IgG antibody level was higher in the i.n. and i.n./i.m. group, which indicated that the combined immunization could induce the stronger mucosal and humoral immune responses. There were no clinical symptoms and no mortality in chickens immunized with the pFNDV-PLGA-NPs i.n. and i.n./i.m after challenge, and the protecting rate was 100% (detailed in Materials and Methods S1).

Despite the recent research progress, the following challenges will need to be addressed in the future. They include trace amounts of initiator, toxic organics and other impurities in the polymer; toxic solvent left in natural polymer during nanoparticles preparation; high cost; and controlled and targeted release of nanoparticles. The solutions are in sight with the advancement of technology in the biomedical sciences and material sciences.

## Supporting Information

Materials and Methods S1 Morphology, size, Zeta potential and encapsulation efficiency measurement of the pFNDV-PLGA-NPs; In vitro release of the pFNDV-PLGA-NPs; Protective efficiency.
(DOC)

Figure S1 **Transmission electron microscopy micrograph of the pFNDV-PLGA-NPs prepared by a double emulsion-solvent evaporation method under the optimized conditions (magnification 10, 000×).**
(TIF)

Figure S2 **Size distribution (A) and Zeta potential (B) of the pFNDV-PLGA-NPs prepared by a double emulsion-solvent evaporation method under the optimized conditions.** A: Measurement of these particles showed a narrow distribution of the pFNDV-PLGA-NPs, and the average diameter was 433.5±7.5 nm; B: Measurement of these particles showed a Zeta potential of +2.7 mV.
(TIF)
