# Supplementary material for: Preparation and Efficacy of Newcastle Disease Virus DNA Vaccine Encapsulated in PLGA Nanoparticles
Source: PLoS One. 2013 Dec 26;8(12):e82648. doi: 10.1371/journal.pone.0082648 (PMC3873271; doi:10.1371/journal.pone.0082648)
Supplement: Materials and Methods S1 — Morphology, size, Zeta potential and encapsulation efficiency measurement of the pFNDV-PLGA-NPs; In vitro release of the pFNDV-PLGA-NPs; Protective efficiency. (DOC) [file pone.0082648.s001.doc]

**Supporting method and material**

***Morphology, size, Zeta potential and encapsulation efficiency measurement of the pFNDV-PLGA-NPs***

Morphology, size, Zeta potential and encapsulation efficiency (EE) measurement of the pFNDV-PLGA-NPs was performed as described previously [22].

***In vitro release of the pFNDV-PLGA-NPs***

The mixture of 0.1 g of the dried pFDNA-PLGA-NPs and 1.0 ml of PBS buffer (pH 7.4) was fully stirred at 4 ^o^C for 200 r/min. One hundred microliters of samples were withdrawn at regular time intervals (0 d, 1 d, 2 d, ……, 15 d), centrifuged at 8000 r/min for 15 min at 4 ^o^C, and the plasmid DNA content in supernatant was analyzed using ultraviolet spectrophotometry. After the sample was collected each time, the same volume was replaced with fresh PBS. The experiment was performed in triplicate. Plasmid DNA release curve of the pFNDV-PLGA-NPs was plotted against the release time at the X-axis and the accumulative release amount at the Y-axis.

***Protective efficiency***

When the level of ND serum antibody of every immune group increased to 6.0 log 2, five chickens were selected at random from the seven groups and infected intramuscularly with the highly virulent NDV strain F_48_E_9_ 1 ml with a viral titer of 10^8^ ELD_50_/0.1 ml for challenge studies. Clinical signs of disease and mortality were monitored on a daily basis, and continuously observed for 14 days.

**Results**

***Characterization of the pFNDV-PLGA-NPs***

The pFNDV-PLGA-NPs prepared by the optimized conditions showed spherical and polydisperse nature as revealed by TEM (Fig. S1). The morphology of the pFNDV-PLGA-NPs had regular round shape and good dispersion, but did not have aggregation or subsidence damage. These particles were measured by a Zeta Sizer 2000 from Malvern Instruments (Southborough, MA, USA) and the average particle size was 433.5 ± 7.5 nm. The particle polydispersity index (PDI) was 0.41 (Fig. S2A) and a zeta potential was +2.7 mV (Fig. S2B).

***In vitro release of the pFNDV-PLGA-NPs***

The in vitro release profiles showed that the release amount of the plasmid DNA from the pFNDV-PLGA-NPs increased quickly from 0 h to 48 h, and the release amount of the plasmid DNA reached 31.25±2.81 % of total plasmid DNA encapsulated in PLGA nanoparticles, and the main cause was due to burst release process of the plasmid DNA from the pFNDV-PLGA-NPs. From 2 to 10 d, the daily release amount of the plasmid DNA reached to 4.53 % of total plasmid DNA encapsulated in the pFNDV-PLGA-NPs following a slow and continuous release, and the process after 10 d was mainly accumulative release of plasmid DNA from the pFNDV-PLGA-NPs. The release amount of the plasmid DNA reached to 93.14 % of total plasmid DNA encapsulated in the pFNDV-PLGA-NPs at 16 d.

***Protective efficacy of the pFNDV-PLGA-NPs***

There were no clinical symptoms and no mortality in chickens immunized with the pFNDV-PLGA-NPs i.n. and i.n. /i.m after challenge, and the protecting rate was 100 %. Pathoanatomical results showed that trachea and cloacae had no hemorrhages and a tiny amount of hemorrhage only appeared in lymphoid follicles of duodenum and cercal tonsil. In chickens immunized with the pFDNA-PLGA-NP i.m and the naked plasmid DNA i.m, one chicken developed the clinical symptoms of lassitude, loss of appetite and then died, and had hemorrhages in iduodenum; the rest of chickens had mild clinical symptoms of lassitude, discharged yellow and green stools, clinical symptoms disappeared in 3 days, and pathoanatomical results showed a tiny amount of hemorrhage in duodenum. Chickens immunized with blank PLGA-NPs and PBS showed the clinical symptoms of lassitude, loss of appetite and even inappetence, discharged yellow and green stools, with the aggravation of state of illness. Paralyzed legs and neck twisted were the major neurological symptoms in some chickens. These chickens died in 3-5 days after the challenge, and the dead chickens had the typical pathological changes of ND such as mucosal hemorrhages in proventriculus papillae, duodenum, heart fat and the whole intestines. The results showed that the pFNDV-PLGA-NPs by intranasal route quickly induced effective mucosal immune response against the challenge.

**Reference**

[22] Zhao K, Shi XM, Zhao Y, Wei HX, Sun QS, Huang TT, *et al*. Preparation and immunological effectiveness of a swine influenza DNA vaccine encapsulated in chitosan nanoparticles. Vaccine 2011; 29: 8549-8556.
