# Supplementary figures and images for: Preparation and Efficacy of Newcastle Disease Virus DNA Vaccine Encapsulated in PLGA Nanoparticles
Source: PLoS One. 2013 Dec 26;8(12):e82648. doi: 10.1371/journal.pone.0082648 (PMC3873271; doi:10.1371/journal.pone.0082648)

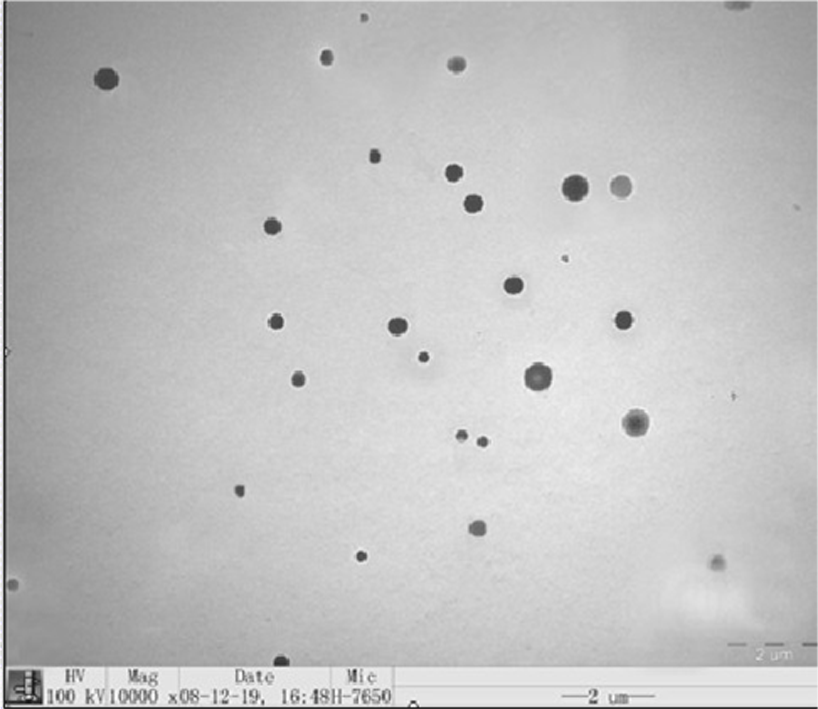

Supplement: Figure S1 — Transmission electron microscopy micrograph of the pFNDV-PLGA-NPs prepared by a double emulsion-solvent evaporation method under the optimized conditions (magnification 10, 000×). (TIF) [file pone.0082648.s002.tif]

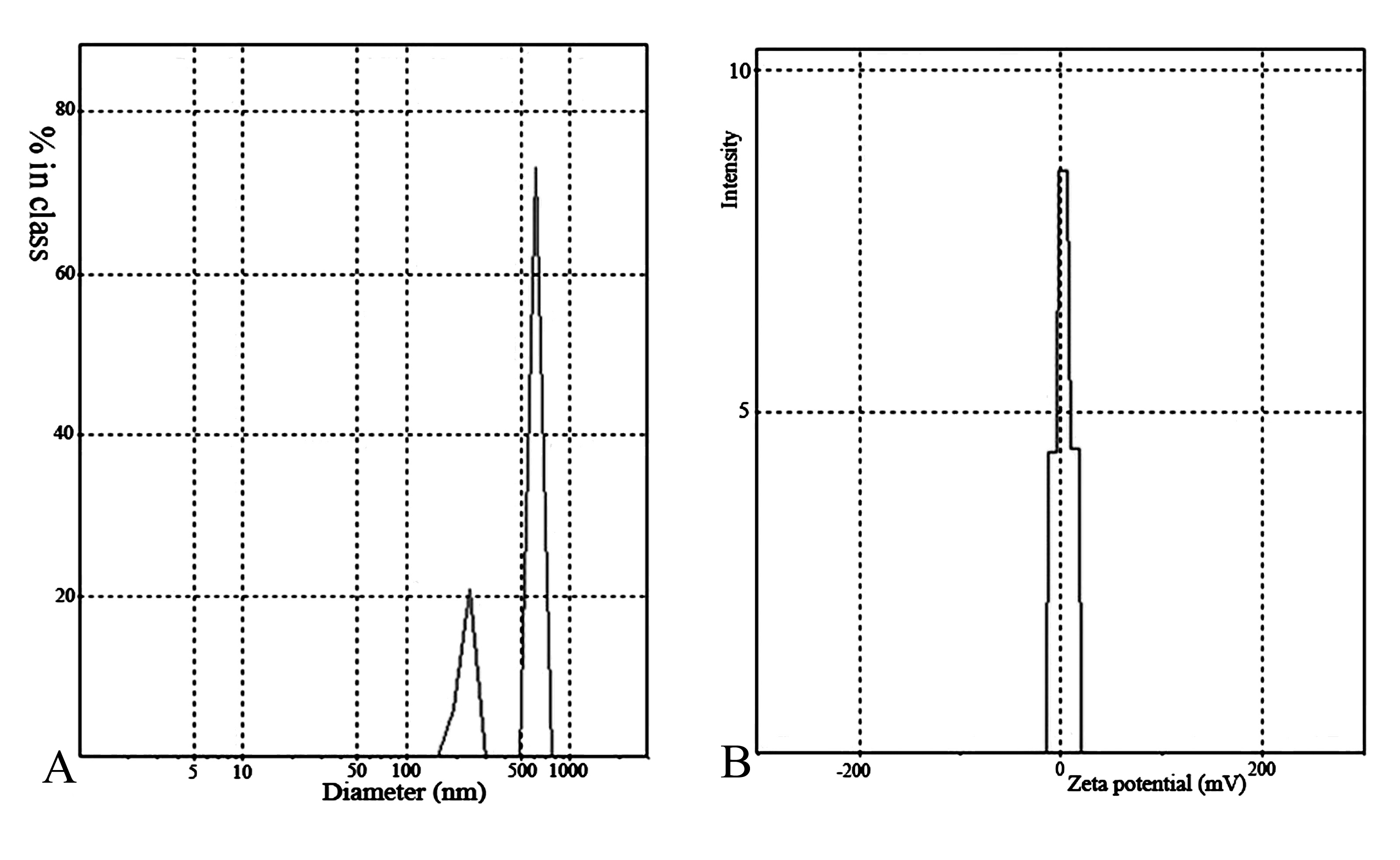

Supplement: Figure S2 — Size distribution (A) and Zeta potential (B) of the pFNDV-PLGA-NPs prepared by a double emulsion-solvent evaporation method under the optimized conditions. A: Measurement of these particles showed a narrow distribution of the pFNDV-PLGA-NPs, and the average diameter was 433.5±7.5 nm; B: Measurement of these particles showed a Zeta potential of +2.7 mV. (TIF) [file pone.0082648.s003.tif]
